# Fulminant liver failure associated with delayed identification of thyroid storm due to heterophile antibodies

**DOI:** 10.1186/s40842-015-0012-6

**Published:** 2015-10-01

**Authors:** Scott A. Soleimanpour

**Affiliations:** Division of Metabolism, Endocrinology & Diabetes and Department of Internal Medicine of the University of Michigan Medical School, 1000 Wall Street, Brehm Tower Suite 5317, Ann Arbor, MI 48105 USA

**Keywords:** Thyrotoxicosis, Human anti-mouse antibodies (HAMA), Hyperbilirubinemia, UDP-glucuronosyltransferase

## Abstract

Hepatic dysfunction during hyperthyroidism frequently occurs with mild abnormalities in liver function tests that are self-limited, improving after treatment of thyroid disease. With the exception of congestive heart failure or secondary hepatic disease, significant hepatic compromise during thyrotoxicosis is rare and often of unexplained origin. This report identifies a novel case of severe hepatic compromise in the setting of thyrotoxicosis that was not initially identified due to a falsely elevated TSH. A 43-year-old African-American man presented to the intensive care unit with severe jaundice, weight loss, thyroid bruit and altered mental status. Initial diagnosis of hyperthyroidism was delayed due to a non-suppressed TSH of 0.20 μU/mL. Laboratory studies identified dramatic hepatic synthetic dysfunction and elevated transaminases with a total bilirubin of 47.4 mg/dL, AST 259 U/L, and ALT 142 U/L. No toxins, structural or viral causes of liver disease were identified and the patient was prepared for potential liver biopsy. Heterophile antibodies were identified and removed by precipitation, demonstrating an undetectable TSH and free thyroxine 9.0 ng/dL consistent with hyperthyroidism. Subsequent treatment with thionamides, corticosteroids, and potassium iodide improved both thyroid and liver function and avoided unnecessary invasive testing. Heterophile antibodies remain as important interfering factors in TSH immunoassays, and thus, this case demonstrates the importance of matching the clinical picture with available laboratory data. In the absence of a known cause of hepatic dysfunction, hyperthyroidism should be considered as a potential etiology of acute liver failure of unknown origin.

## Background

Mild hepatic dysfunction is common during hyperthyroidism and is most frequently associated with abnormalities in liver function tests [1, 2]. While mild elevations in serum alkaline phosphatase values are most frequently associated with hyperthyroidism, severe hepatic dysfunction complicated by jaundice has been rarely found in cases of severe thyrotoxicosis [3–7]. Most often, alterations in serum liver tests are self-limited and resolve following treatment and improvement of hyperthyroidism [1, 2].

Significant hepatic dysfunction and evidence of liver failure is an infrequent consequence of hyperthyroidism, most frequently associated with congestive heart failure or underlying secondary hepatic dysfunction [7]. This report identifies a novel case of severe hepatic compromise in the setting of thyrotoxicosis, which was potentially exacerbated due to a delayed diagnosis of thyrotoxicosis secondary to heterophilic antibody interference with the thyrotropin (TSH) assay.

## Case presentation

A 43-year-old homeless African-American man presented to the intensive care unit with severe jaundice and altered mental status. The patient had an unintentional 100 lb. weight loss over the previous 6 months, along with rapid heart rate, extreme fatigue, and frequent diarrhea. Two weeks prior to admission, the patient was evaluated for nausea, vomiting, jaundice, and dehydration and was discharged home to arrange outpatient follow-up of an unspecified hepatic and thyroid defect. He had no previous history of liver disease, exposure to jaundiced people or hepatitis, exposure to hepatotoxins (such as acetaminophen), alcohol ingestion, or drug abuse and was not taking any medications. Physical examination revealed an ill-appearing, cachectic man with temporal wasting who was unresponsive to external stimuli. His pulse was 150 beats/min and irregular; blood pressure 140/70 mmHg, temperature 36.7 °C, and weight 146 lb. Sclerae were icteric, but without proptosis or exophthalmos. His thyroid gland was diffusely enlarged (40 g by palpation) with a palpable thyroid thrill and audible thyroid bruit. There were no palpable thyroid nodules. Cardiovascular examination revealed an irregular rate and rhythm, no jugular venous distension, and no murmurs. The patient had a tender right upper quadrant on palpation of his abdomen, with hepatomegaly palpable 4–5 cm below the costal margin and no ascites. Neurologic examination revealed a resting tremor with hyperreflexia.

Laboratory data was remarkable for an elevated total bilirubin of 47.4 mg/dL (normal range: 0.2–1.2 with direct bilirubin of 44.6 mg/dL, delta bilirubin of 18.5 mg/dL, conjugated bilirubin of 26.1 mg/dL, unconjugated bilirubin of 2.8 mg/dL); aspartate aminotransferase of 259 U/L (AST; normal range: 8–60); alanine aminotransferase 142 U/L (ALT: normal range 14–78), alkaline phosphatase 307 U/L (normal range: 38–126 U/L); albumin 3.3 mg/dL (normal range 3.5–5.0); and prothrombin time (PT) >90 s. Complete blood count, platelets, and reticulocyte count were within reference ranges. Blood and urine toxicology (including alcohol), hepatitis A/B/C serologies, antinuclear antibodies, anti-mitochondrial antibodies, anti-gliadin antibodies, and HIV serology results were negative. Serum haptoglobin, ceruloplasmin, CEA, CA 19–9, AFP measurements were within normal limits. Electrocardiogram revealed atrial fibrillation and echocardiogram demonstrated tachycardia, preserved cardiac function with an ejection fraction of 70 %, and no evidence of congestive heart failure. Abdominal CT imaging revealed hepatomegaly without evidence of focal hepatic mass, inflammation, or vascular obstruction. The lack of a specific diagnostic cause for fulminant liver failure led to planning for hepatic biopsy to determine a potential pathologic liver defect.

Thyroid function studies revealed a thyroid-stimulating hormone of 0.20 μU/mL (TSH; normal range: 0.4–4.0); total thyroxine 15.2 μg/dL (total T4; normal range 4.5–12.5); free thyroxine 9.0 ng/dL (free T4; normal range 0.89–1.80); T3 uptake 1.98 (normal range 0.9–1.3 TBI); and total triiodothyronine 3.2 ng/mL (total T3; normal range 0.6–1.71). The patient’s history of homelessness as well as reported contact with rodents (and thus rodent antigens) raised the suspicion that measured TSH values were falsely elevated due to heterophile antibody interference with the TSH assay. Heterophile antibodies consist of natural antibodies that are weak and polyspecific antibodies capable of interference with immunoassays, such as TSH [8]. Following removal of human anti-mouse antibodies (HAMA) by precipitation, subsequent measurement of TSH concentration was <0.01 mU/L. Furthermore, a screen for HAMA was positive.

**Fig. 1:**
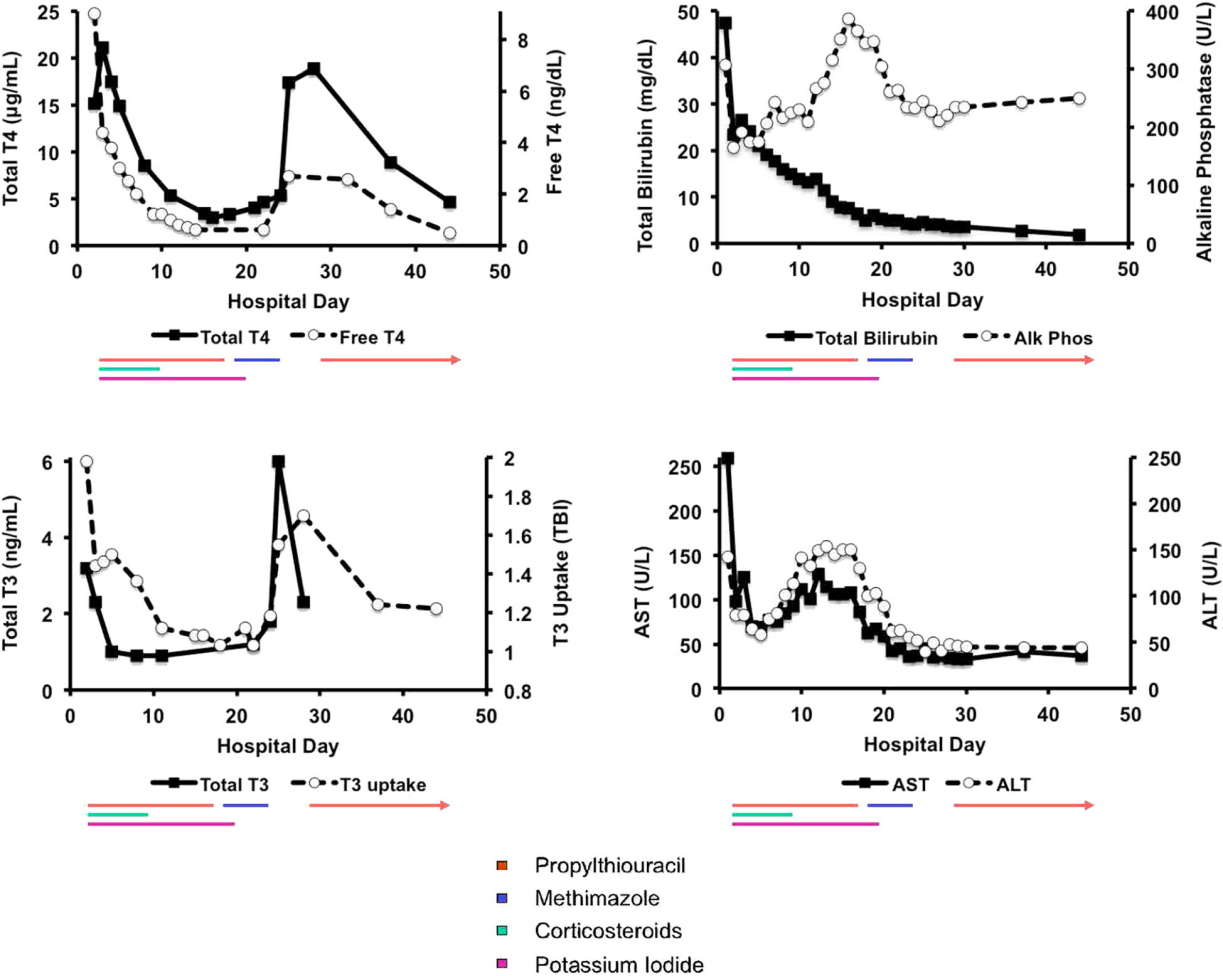
Both liver and thyroid function studies improve following treatment for hyperthyroidism. Total T4 (reference range 4.5–12.5 μg/mL), free T4 (reference range 0.89–1.80 ng/dL), total bilirubin (reference range 0.2–1.2 mg/dL), alkaline phosphatase (reference range 38–126 U/L), total T3 (reference range 0.6–1.71 ng/dL), T3 uptake (reference range 0.90–1.30 TBI) AST (reference range 8–60 U/L), ALT (reference range 14–78 U/L) levels measured over hospital course during therapy with propylthiouracil (PTU; orange), methimazole (MMI; blue), corticosteroids (dexamethasone; green), and potassium iodide (SSKI; purple). PTU 400 mg q6 h, dexamethasone, and SSKI 4 drops q6 h were started on the end of day 2 of hospitalization. Dexamethasone was discontinued and PTU was reduced to 200 mg q8 h on day 9. SSKI was reduced to 2 drops q8 h on day 12. PTU was discontinued and MMI 40 mg daily was initiated on day 13. MMI and SSKI were discontinued on days 16 and 18, respectively. PTU was then restarted at 200 mg q8 h on day 25 and maintained at 150 mg q8 h after day 37 of hospitalization

Based on previously published diagnostic guidelines [9], a clinical and laboratory diagnosis of thyroid storm due to Graves’ disease was made and treatment with propylthiouracil (PTU; 400 mg every 6 h), potassium iodide, corticosteroids, and beta blockers was initiated by the end of the second day of hospitalization. After 24 h of treatment, free T4 and total T3 levels began to decline, while total T4 levels rose, potentially related to the acute inhibitory activity of PTU on type 1 deiodinase [10, 11]. Within 2–3 days of therapy, liver function studies began to improve, along with improvement of T4 levels. By day 5 of therapy, mental status and functioning improved. Corticosteroids were discontinued within 10 days of treatment. Scleral icterus and jaundice resolved by day 15 of treatment. From days 10–25, thyroxine levels dropped below normal control concentrations, and thionamides and iodide were briefly discontinued. Of note, thionamide therapy with PTU was initiated at the outset of treatment, but briefly substituted with methimazole due to concern over hepatic toxicity. These concerns were ultimately unfounded, as a lower dosage of PTU (200 mg every 8 h) was reintroduced by one month after treatment initiation and was maintained with dramatic improvement of total bilirubin, AST, and ALT levels (detailed in Fig. 1). Hepatic synthetic activity also dramatically improved as prothrombin time and albumin concentrations returned to reference ranges within 2–3 weeks. Alkaline phosphatase concentrations improved by one month of treatment, but not completely to normal ranges, prompting an evaluation of bone and liver isoenzymes, which were both elevated; bone-specific alkaline phosphatase of 88 IU/L (normal range: 0–55) and liver-specific alkaline phosphatase of 278 IU/L (normal range: 16–70) by 2 weeks of therapy. By 4 weeks after treatment, the patient was moved to an inpatient rehabilitation facility, where he subsequently gained weight and improved his functional conditioning while continuing PTU therapy. Prior to discharge from the hospital, an I-123 uptake and nuclear scan was performed revealing diffuse 74 % uptake at 2 h and 43 % uptake at 24 h. He was subsequently treated with 18.7 mCi of I-131 for ablative therapy.

Given his severe liver failure during thyrotoxicosis, previous studies have suggested that an underlying defect in bilirubin metabolism could be aggravated by thyrotoxicosis [12, 13]. In order to test for the presence of genetic mutations affecting bilirubin metabolism, genomic DNA was extracted from peripheral blood using a commercially available kit (Genetra systems, Inc.) and followed by automated DNA sequencing [14] at a reference laboratory at the Children’s Hospital of Philadelphia. The patient was found to be heterozygous for Gilbert’s syndrome, an autosomal recessive disorder, which is characterized by mild, unconjugated hyperbilirubinemia in the absence of liver disease or hemolysis. Gilbert’s syndrome is due to the presence of genetic defects in the promoter region of the *UGT1A1* gene, which encodes the UDP-glucuronosyltransferase enzyme and is a key component for efficient biliary excretion of bilirubin [15]. Patients with Gilbert’s syndrome possess an additional TA repeat in the TATAA element of the 5′ promoter of the gene ((TA)_7_TAA rather than the normal (TA)_6_TAA), but patients with heterozygous mutations for Gilbert’s syndrome are often asymptomatic and do not exhibit hyperbilirubinemia. Furthermore, the patient did not have suppression or absence of albumin-bound delta bilirubin, a phenomenon reported during hyperbilirubinemia secondary to hemolysis or Gilbert’s syndrome [16].

## Conclusions

This patient experienced severe hepatic dysfunction in the setting of Graves’ disease and thyroid storm. Unique to this case was the presence of heterophile antibodies to the TSH assay, which led to a delayed diagnosis and treatment of hyperthyroidism, as a thyroid-derived liver defect was not initially considered. Changes in thyroid laboratory tests secondary to critical illness [17, 18], otherwise known as sick euthyroid syndrome, were considered in the patient’s differential diagnosis but appeared less likely despite the overt pathognomonic signs of hyperthyroidism and dramatically elevated total and free T4 values. Furthermore, TBG excess (with reduced T3 resin uptake) secondary to acute hepatitis was considered as a potential etiology for the patient’s thyroid laboratory abnormalities [19, 20], but this possibility seemed less likely as the patient displayed an elevation in his T3 uptake. Co-existing autoimmune liver diseases (autoimmune hepatitis or primary biliary cirrhosis) were also considered as potential causes of hepatic dysfunction, but these diagnoses seemed less likely due to the absence of ANA or anti-mitochondrial antibodies as well as the acuity of his presentation and his laboratory results [21]. Moreover, the subsequent treatment of thyrotoxicosis not only improved thyroid status, but also resolved liver dysfunction, which would not be anticipated with sick euthyroid syndrome, TBG excess, or autoimmune liver disease.

TSH measurements are a critical component in the diagnosis of thyroid disorders. While 3^rd^ generation TSH immunoassays offer substantial sensitivity in the determination of thyroid function, these immunoassays can still suffer from interference from heterophile antibodies [22]. The generalized incidence of heterophile antibodies is variable but has been reported to be as high as 10.4–11.7 % of all patients, with an increased risk for development in patients who have received animal-derived pharmaceutical immunoglobulin therapies, blood transfusions, vaccinations, as well as naturally encountered antigens from pets or due to animal husbandry [23, 24]. Given this patient’s exposure to rodent antigens while homeless, it is possible that the formation of HAMA led to the falsely elevated TSH that delayed his diagnosis. The aberrant elevation of this patient’s TSH concentrations serves as a reminder of the importance of ensuring the clinical picture matches available laboratory data to prevent unnecessary testing, procedures, or morbidity.

Liver dysfunction is a common occurrence in hyperthyroidism, but its etiology is not well known. Previous histopathologic analyses note diverse changes in the livers of patients with hyperthyroidism-induced hepatic dysfunction, ranging from intracellular cholestasis, inflammation with hepatocellular damage, to overt cirrhosis [2, 25]. Interestingly, pre-clinical models demonstrate that hyperthyroidism can impair UDP glucuronosyltransferase activity and to lead to elevated bilirubin levels [13]. Furthermore, a previous report has observed cases of jaundice with thyrotoxicosis in patients with latent defects in UDP-glucuronosyltransferase activity due to Gilbert’s disease [12]. While we could not measure UDP- glucuronosyltransferase activity in this patient, it should be noted that patients with heterozygous mutations for Gilbert’s disease do not normally exhibit hepatic dysfunction, and this patient did not appear to have any other known secondary cause (such as CHF or underlying liver disease) to precipitate hepatic compromise. While routine genetic screening may not always be indicated for patients with hyperthyroidism and concomitant severe liver disease, future studies will be invaluable to determine the molecular mechanisms of severe hepatic compromise in patients with thyroid storm.

Elevated alkaline phosphatase levels are a common consequence following the resolution of hyperthyroidism, likely due to elevations in bone-specific alkaline phosphatase [3]. Serum alkaline phosphatase levels often take several months to improve following resolution of hyperthyroidism, as enhanced bone turnover likely persists [1, 26]. These observations are consistent with this patient’s presentation, as both bone and liver specific alkaline phosphatase levels continued to be elevated within weeks of therapy.

In summary, this is a novel case of severe hepatic dysfunction in the setting of thyrotoxicosis compounded by the presence of heterophile antibodies. Importantly, early identification and treatment of Graves’ disease based upon his clinical signs and symptoms, rather than laboratory studies alone, may have prevented severe morbidity due to delayed treatment. Additionally, in patients with thyrotoxicosis and subsequent hyperbilirubinemia, treatment of thyroid dysfunction with thionamides is safe and effective resulting in improved liver function. These findings may be of benefit to endocrinologists in understanding liver disease in thyrotoxic patients or to gastroenterologists in the identification of the etiology of acute liver failure of unknown origin.

## Consent

Informed consent was obtained from the patient for publication of this case report. A copy of the written consent is available for review on request.
